# Asymptomatic Pneumoperitoneum With a Large Amount of Gas Appeared During Endoscopic Ultrasound-Guided Biliary Drainage

**DOI:** 10.7759/cureus.54330

**Published:** 2024-02-16

**Authors:** Koji Takahashi, Hiroshi Ohyama, Izumi Ohno, Yuichi Takiguchi, Naoya Kato

**Affiliations:** 1 Department of Gastroenterology, Chiba University, Chiba, JPN; 2 Department of Medical Oncology, Chiba University, Chiba, JPN

**Keywords:** stent, pneumoperitoneum, endoscopic ultrasound-guided rendezvous, endoscopic ultrasound-guided hepaticogastrostomy, endoscopic ultrasound-guided biliary drainage

## Abstract

We report a case in which a large amount of intraperitoneal free gas developed during endoscopic ultrasound-guided biliary drainage with the rendezvous technique. A 62-year-old woman presented with obstructive jaundice caused by a pancreatic head tumor. Endoscopic retrograde cholangiopancreatography was attempted but failed due to difficulty cannulating the bile duct. Consequently, endoscopic ultrasound-guided hepaticogastrostomy was performed using a fully covered metal stent. Subsequently, the rendezvous technique was employed to access the biliary system and perform an endoscopic sphincterotomy. Finally, a fully covered metal stent was placed transpapillary. Fluoroscopic imaging during the procedure revealed a large amount of gas between the liver and diaphragm. Despite the pneumoperitoneum, the patient experienced no abdominal pain or fever. One week later, a computed tomography scan confirmed the disappearance of free air in the intraperitoneal cavity. The patient’s subsequent clinical course remained uneventful, and she was discharged from the hospital. This case highlights the potential for pneumoperitoneum to develop during endoscopic ultrasound-guided biliary drainage, particularly when using the rendezvous technique. It is crucial to differentiate this finding from gastrointestinal perforation based on clinical presentation and imaging features.

## Introduction

Endoscopic retrograde cholangiopancreatography (ERCP) is the first-line treatment for biliary drainage in cases of obstructive jaundice and acute cholangitis [1]. ERCP-based biliary drainage methods include biliary stenting and endoscopic nasobiliary drainage. However, in some cases, biliary drainage using ERCP is difficult for various reasons, necessitating alternative biliary drainage procedures using endoscopic ultrasonography (EUS). Endoscopic ultrasound-guided biliary drainage (EUS-BD) is a widely performed procedure with numerous reports demonstrating its clinical efficacy [2]. Nevertheless, due to its shorter history compared to ERCP, much remains unknown regarding its adverse events. Because EUS-BD involves puncturing the bile duct from the gastrointestinal tract, a small amount of gas may appear in the abdominal cavity after the procedure. However, large amounts of intraperitoneal gas rarely appear without symptoms. Here, we report a case where a large amount of intraperitoneal free gas developed during a combined procedure involving an endoscopic ultrasound-guided hepaticogastrostomy (EUS-HGS) and an endoscopic ultrasound-guided rendezvous (EUS-RV) technique.

## Case presentation

**Figure 1:**
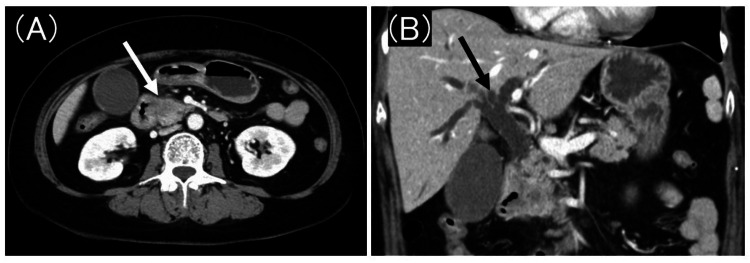
Contrast-enhanced computed tomography (A) Contrast-enhanced computed tomography scan demonstrates a pancreatic head tumor (white arrow) with duodenal invasion. (B) The bile duct (black arrow) upstream of the tumor is significantly dilated.

A 62-year-old woman presented with abdominal discomfort at a local hospital. She had a history of hypertension and left breast cancer treated with a left partial mastectomy. Abdominal ultrasound revealed a tumor in the pancreatic head, prompting referral to our hospital for further evaluation. Blood tests confirmed elevated hepatobiliary enzymes and jaundice., while a contrast-enhanced computed tomography (CT) scan revealed a pancreatic head tumor with duodenal invasion. The upstream bile duct appeared dilated (Figure 1). Based on these findings, the patient was diagnosed with obstructive jaundice due to a pancreatic head tumor.

**Figure 2:**
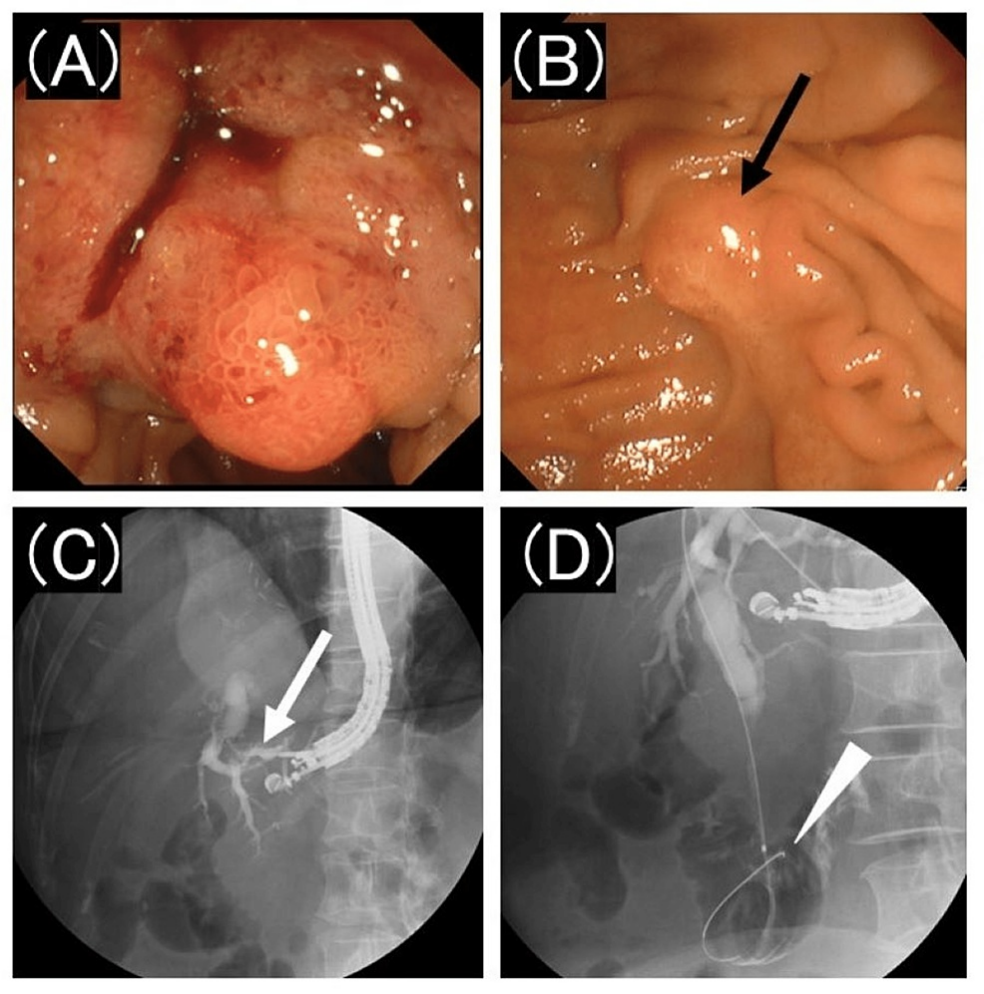
Image of the duodenum during endoscope insertion and fluoroscopic image during endoscopic ultrasound-guided hepaticogastrostomy (A) The pancreatic tumor invades the duodenum. (B) The endoscope successfully reaches the duodenal papillae (black arrow). Then, attempts to insert a catheter into the bile duct prove unsuccessful. (C) An echoendoscope is inserted, and the B3 segment of the intrahepatic bile duct (white arrow) is punctured transgastrically with a 19-gauge needle. The guidewire is then inserted into the bile duct. (D) The guidewire (arrowhead) is easily guided through the duodenal papillae and into the duodenal lumen.

Initial attempts at biliary drainage using ERCP were made. Carbon dioxide insufflation was used during this procedure. Although the tumor had invaded the duodenum, the endoscope reached the duodenal papillae. However, inserting a catheter into the bile duct proved impossible. Consequently, we opted for EUS-BD on the same day. Using a linear echoendoscope (GF-UCT260; Olympus, Tokyo, Japan), we successfully punctured the B3 segment of the intrahepatic bile duct transgastrically with a 19-gauge needle (EZ shot 3 plus; Olympus, Tokyo, Japan), and inserted a guidewire (VisiGlide 2; Olympus, Tokyo, Japan) into the bile duct. The bile duct was punctured only once during the procedure. The Seldinger technique was not used for bile duct puncture, and the guidewire did not exit the abdominal cavity. The guidewire can be easily navigated through the duodenal papillae and into the duodenal lumen (Figure 2). The plan was made to insert two biliary stents: one for hepaticogastrostomy and the other across the duodenal papillae. In one method, the stent was placed antegradely across the duodenal papillae from the puncture site using EUS. However, we believe that placing a stent using an EUS-guided antegrade technique would increase the possibility of pancreatitis because endoscopic sphincterotomy (EST) would not be possible. Pancreatitis can be fatal; therefore, we aimed to reduce its risk. To reduce the risk of postprocedural pancreatitis, we planned to use the EUS-RV technique, which involves EST followed by stent placement via ERCP instead of the EUS-guided antegrade technique.

**Figure 3:**
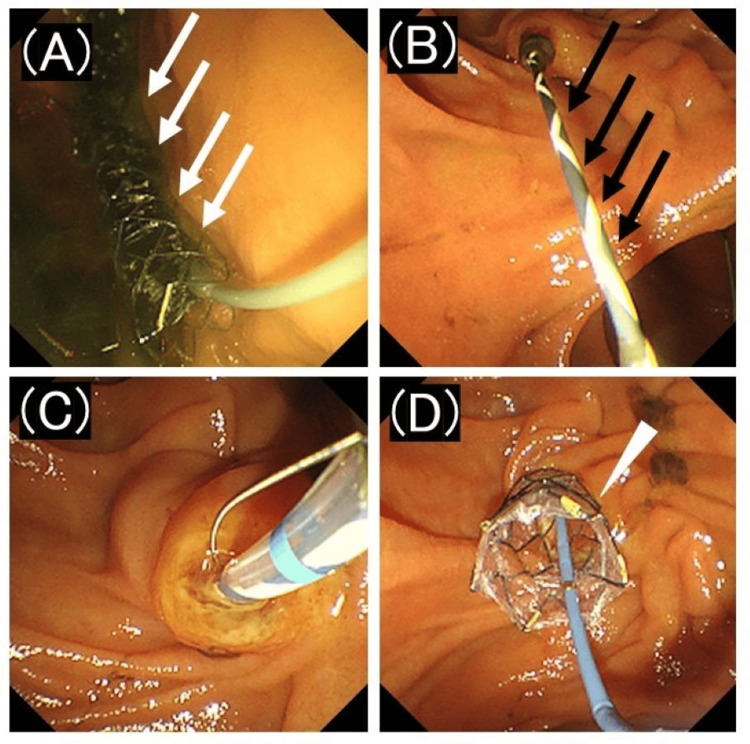
Endoscopic images of endoscopic ultrasound-guided hepaticogastrostomy and endoscopic ultrasound-guided rendezvous technique (A) A fully-covered metal stent (white arrow) with an 8 mm diameter is placed between the intrahepatic bile duct and the stomach. (B) To perform endoscopic retrograde cholangiopancreatography using the rendezvous technique, the guidewire (black arrow) positioned in the duodenal lumen is grasped and pulled into the endoscope. (C) An endoscopic sphincterotomy is performed before stent placement. (D) A fully covered metal stent (arrowhead) with a diameter of 10 mm is placed transpapillary in the extrahepatic bile duct.

An MTW ERCP catheter (MTW Endoskopie Manufaktur; Wesel, Germany) was inserted into the bile duct through the stomach wall, and additional bile duct imaging with contrast medium was performed. Subsequently, without additional dilation of the puncture site, a fully covered metal stent (HANAROSTENT Biliary Full Cover Benefit; Boston Scientific, Natick, MA, USA), 8 mm in diameter, was placed between the intrahepatic bile duct and the stomach. Subsequently, ERCP was performed using the rendezvous technique. The guidewire, positioned in the duodenal lumen, was grasped and pulled into the endoscope. The EST was performed using a sphincterotome (CleverCut3V; Olympus, Tokyo, Japan) and transpapillary placement of a 10-mm fully covered metal stent (HANAROSTENT Biliary Full Cover NEO; Boston Scientific, Natick, MA, USA) in the extrahepatic bile duct (Figure 3).

**Figure 4:**
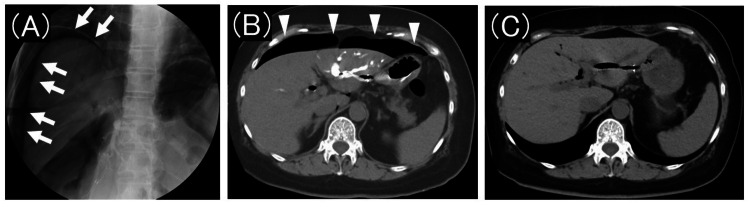
Fluoroscopic and computed tomography images of pneumoperitoneum (A) During endoscopic retrograde cholangiopancreatography, a large amount of gas (arrow) appeared between the liver and diaphragm on fluoroscopic images. (B) A computed tomography scan immediately after endoscopic retrograde cholangiopancreatography reveals a substantial amount of free air (arrowhead) within the peritoneal cavity. No intraperitoneal leakage of the contrast medium is observed. (C) A computed tomography scan conducted one week after the procedure demonstrates complete resolution of the free air within the peritoneal cavity.

Fluoroscopic images showed no intraperitoneal free air at the start of ERCP but a significant amount of gas between the liver and diaphragm during ERCP. A post-procedure CT scan confirmed the presence of a large amount of free air within the peritoneal cavity (Figure 4). No intraperitoneal leakage of the contrast medium was observed.

Given the absence of abdominal pain, fever, or signs of peritoneal irritation, we concluded that the gas originated from the stomach during the procedure. We opted for conservative management with close monitoring. The patient tolerated oral intake without any subjective symptoms, and a repeat CT scan conducted one week later demonstrated complete resolution of the free air in the peritoneal cavity. A biopsy obtained from the tumor-invaded portion of the duodenum confirmed the histopathological diagnosis of pancreatic adenocarcinoma.

Following the improvement in her jaundice, the patient underwent contrast-enhanced magnetic resonance imaging. This revealed the presence of multiple liver metastases. Based on these findings, we diagnosed the patient with pancreatic head cancer and liver metastases and planned to initiate systemic therapy. After discharge, the patient was started on gemcitabine and nab-paclitaxel chemotherapy. There has been no recurrence of pneumoperitoneum to date.

## Discussion

In this case, the patient underwent ERCP using the EUS-RV technique following EUS-HGS. While a significant amount of intraperitoneal free gas was observed during the procedure, the patient experienced no specific symptoms, and the gas gradually dissipated. This suggests the gas leak was temporary and did not persist after the procedure, implying no ongoing communication between the gastrointestinal tract and the abdominal cavity.

EUS-guided cholangiography was first reported in 1996 [3]. EUS-BD techniques include endoscopic ultrasound-guided choledochoduodenostomy (EUS-CDS), EUS-HGS, and EUS-RV. The initial reports for these techniques were published in 2001 [4] (EUS-CDS), 2003 [5] (EUS-HGS), and 2004 [6] (EUS-RV), respectively. Each EUS-BD technique has its own unique profile of potential adverse events. Some of the more common adverse events associated with EUS-CDS include biliary leakage, stent migration, hemorrhage, perforation, and bile peritonitis [5-7]. For EUS-HGS, potential adverse events include hemorrhage, bile leakage, biloma, stent migration, stent misplacement, intrahepatic hematoma, and sepsis [7-9]. In the case of EUS-RV, common adverse events include acute pancreatitis, pneumoperitoneum, bile peritonitis, and hemorrhage [10,11].

In 2018, clinical practice guidelines for EUS-BD were published in Japan. This report documented the incidence of pneumoperitoneum as 0 of 144 EUS-CDS procedures, 0 of 247 EUS-HGS procedures, and 8 of 364 EUS-RV procedures (2.2 %) [12]. These findings suggest that pneumoperitoneum is unlikely to occur in EUS-CDS or EUS-HGS but rather constitutes an adverse event specific to EUS-RV. EUS-HGS and EUS-CDS typically involve minimal air or carbon dioxide insufflation, whereas EUS-RV requires a certain amount of gas for ERCP. This suggests that the insufflated carbon dioxide gas may leak into the peritoneal space from the puncture site during EUS-RV, contributing to its higher incidence of pneumoperitoneum.

The addition of antegrade stent placement to EUS-HGS can reportedly prolong the time to stent dysfunction and reduce adverse events related to bile leakage [13,14]. However, EUS-HGS alone can provide sufficient drainage to improve jaundice regardless of whether a metal or plastic stent is used [15]. Moreover, there is a risk of pancreatitis associated with antegrade stent placement. EST effectively reduces the risk of pancreatitis, but it cannot be performed using the antegrade stent placement technique. Therefore, we chose EUS-RV over antegrade stent placement and performed EST. However, although pancreatitis did not occur, pneumoperitoneum developed. If transpapillary stenting is required to ensure long-term stent patency, EUS-RV after fistula formation would prevent pneumoperitoneum. In addition, pushing the scope into the duodenum immediately after EUS-HGS stent replacement carries the risk of stent migration or dislocation. In particular, caution is required when puncturing the B3 segment of the intrahepatic bile duct. Puncturing the B3 segment in EUS-HGS is performed at a distance from the puncture site to the bile duct, so it may be better not to do so in cases where EUS-HGS followed by EUS-RV is intended.

It is suggested that pneumoperitoneum may be a more frequent occurrence with EUS-RV compared to other EUS-BD techniques, highlighting the importance of awareness and vigilance during such procedures. Differentiating pneumoperitoneum from gastrointestinal perforation is crucial in such situations. Careful assessment of patient symptoms and examination findings, including the absence of fever, abdominal pain, and signs of peritoneal irritation, can guide the diagnosis and appropriate management decisions. Overall, this case highlights the need for further investigation into the potential increased risk of pneumoperitoneum with combined EUS-HGS and EUS-RV procedures. Additionally, close monitoring of patients and prompt evaluation of any suggestive symptoms are essential to ensure optimal patient care.

## Conclusions

We report a case of a large amount of intraperitoneal free gas following a combined EUS-HGS and EUS-RV procedure, despite the absence of associated symptoms. This case highlights the potential for pneumoperitoneum to develop during endoscopic ultrasound-guided biliary drainage, particularly when using the rendezvous technique. It is crucial to differentiate this finding from gastrointestinal perforation based on clinical presentation and imaging features.
